# Three differently coloured polymorphs of 3,6-bis­(4-chloro­phenyl)-2,5-di­propyl-2,5-di­hydro­pyrrolo­[3,4-*c*]pyrrole-1,4-dione

**DOI:** 10.1107/S2052520619004773

**Published:** 2019-05-23

**Authors:** Hee-Soo So, Shinya Matsumoto

**Affiliations:** a Graduate School of Environment and Information Sciences, Yokohama National University, 79-7 Tokiwadai, Hodogaya-ku, Yokohama, 240-8501, Japan

**Keywords:** conformational polymorph, diketo­pyrrolo­pyrrole, propyl substituent, halogen-bonding interaction

## Abstract

In this paper, the conformational polymorphism of a chlorinated diketo­pyrrolo­pyrrole (DPP) dye having flexible substituents in a non-hydrogen-bonding system is reported. The propyl-substituted DPP derivative (PR3C) has three polymorphic forms, each showing a different colour (red, orange and yellow). All polymorphs could be obtained concomitantly under various crystallization conditions. The results of the crystal structure analysis indicate that PR3C adopts different conformations in each polymorph. The packing effect caused by the difference in the arrangement of neighbouring molecules was found to play an important role in the occurrence of the observed polymorphism. The thermodynamic stability relationship between the three polymorphs was identified by thermal analysis and indicates that the yellow polymorph is the thermally stable form. The results indicate that the yellow form and orange form are enantiotropically related, and the other polymorph is monotropically related to the others.

## Abstract 2

A very rare example of a diketo­pyrrolo­pyrrole derivative showing polymorphism with completely different molecular conformations and a different arrangement of neighbouring molecules is reported. The thermodynamic stability relationships between the three polymorphic forms are also interpreted.

## 1. Introduction   

Polymorphism refers to the phenomenon where a compound has two or more crystal structures for the same chemical substance (Bernstein, 2002 ▸). Different polymorphs play an important role in the pharmaceutical, food and pigment industries because they exhibit different physicochemical properties in the crystalline state (Borka & Haleblian, 1990 ▸; Threlfall, 1995 ▸; Zollinger, 2001 ▸; Inabe & Tajima, 2004 ▸; Le Révérend *et al.*, 2010 ▸; Hunger & Schmidt, 2018 ▸). Therefore, a method for controlling the occurrence of polymorphs is required, and considerable efforts have been made to characterize different polymorphs (Threlfall, 1995 ▸; Miller *et al.*, 2005 ▸; Pellizzeri *et al.*, 2014 ▸).

Important factors affecting conformational polymorphism which results from the different conformations of the same molecule are the number of torsional degrees of freedom of the molecular structure and the introduction of flexible substituents (Nangia, 2008 ▸; Cruz-Cabeza & Bernstein, 2014 ▸). In this respect, our group has shown that polymorphs of a series of 2,5-di­amino-3,6-di­cyano­pyrazine derivatives can be induced by conformational changes arising from the flexibility of the di­benzyl groups substituted at its two amino positions (Matsumoto *et al.*, 2006 ▸). Recently, in this series of pyrazine derivatives, systematic studies into the correlation of halogen-bonding interactions (*X*⋯*X*, *X*⋯heteroatom and C—H⋯*X*) with the occurrence of polymorphs have been made; this was achieved by introducing several halogen atoms at the *ortho* and *para* positions of the benzyl group (Akune *et al.*, 2015 ▸; Akune *et al.*, 2016 ▸; Akune, Hirosawa, Koseki *et al.*, 2017 ▸; Akune, Hirosawa, Endo *et al.*, 2017 ▸). Among the reported derivatives, the weak halogen-bonding interactions via the electrostatic properties of Cl and Br atoms were found to be particularly important for the structural diversity in these pyrazine dyes, and the polymorphs were dependent on the substituent position.

**Figure 1:**
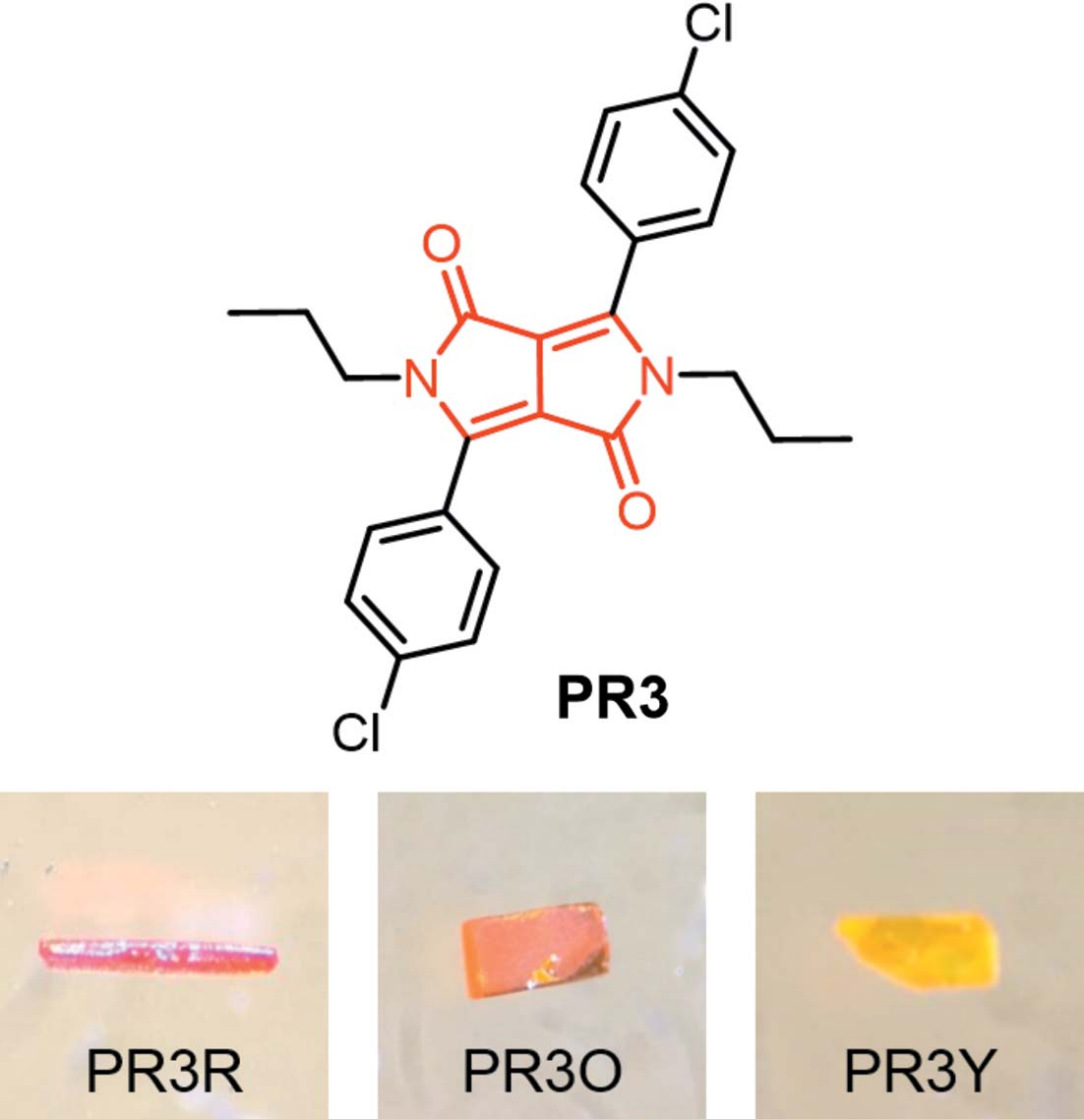
Chemical structure and three differently coloured polymorphs of PR3C. The red part in the structural formula represents the DPP core.

Diketo­pyrrolo­pyrroles (DPP) are an important class of organic pigments (Iqbal, Jost *et al.*, 1988 ▸; Iqbal, Cassar *et al.*, 1988 ▸) and have been studied as functional dyes in opto-electronic applications including materials for organic light-emitting diodes, semiconductors and solar cells (Mei *et al.*, 2011 ▸; Liu *et al.*, 2013 ▸; Printz *et al.*, 2014 ▸; Gasperini *et al.*, 2015 ▸; Shin *et al.*, 2015 ▸; Data *et al.*, 2016 ▸). Also, 3,6-bis (4-chloro­phenyl)-DPP (C.I. Pigment Red 254) is widely used today as a high-performance pigment for automotive and industrial paint applications (Wallquist & Lenz, 2009 ▸). Unlike the increasing number of reports on these applications of DPP derivatives, the number of reports into their polymorphs has been limited (Langhals *et al.*, 1989 ▸; MacLean *et al.*, 2000 ▸; Mizuguchi, 2003*a* ▸,*b* ▸; MacLean *et al.*, 2009 ▸; Calvo-Castro *et al.*, 2014 ▸). Calvo-Castro *et al.* reported that an *N*,*N*′-bis­benzyl­ated chlorinated DPP derivative exhibited two polymorphic forms, and they indicated that the role of the substituents, both halogen atoms and benzyl groups, was important in the displacement of the molecules of each polymorphic form in the crystal structure. However, the effect of the intermolecular interactions of the Cl atoms on the polymorphism has not been fully discussed. On the other hand, systematic research into di­amino­dicyano­pyrazine dyes by Akune *et al.* indicated that weak intermolecular interactions with respect to Cl and Br atoms are very important for the occurrence of polymorphism, as mentioned above. We were, thus, motivated to attempt to identify the effects of Cl atoms and flexible substituents on the polymorphism of compounds with the DPP chromophore with respect to its potential for a wide range of functional applications. In our earlier study on the occurrence of polymorphism of *N*,*N*′-bis­propyl­ated chlorinated-DPP dye [3,6-bis­(4-chloro­phenyl)-2,5-di­propyl-2,5-di­hydro­pyrrolo[3,4-*c*]pyrrole-1,4-dione, hereafter abbreviated as PR3C], we found two polymorphs; furthermore, the yellow polymorph (PR3Y) was found to change into the orange polymorph (PR3O) with dynamical behaviour such as bursting, flipping and jumping upon heating. We determined that this dynamical behaviour might be associated with a sudden release of accumulated stress on one propyl group upon heating (So *et al.*, 2018 ▸). We also obtained a new red polymorph (PR3R) in addition to PR3O and PR3Y (Fig. 1 ▸). In the present report, their molecular and crystal structures are carefully examined and we analyse and interpret the effect of the above two substituents on the intermolecular interactions using theoretical methods. The thermal relationship between the three polymorphs is also identified using thermal analysis.

## 2. Experimental   

### 2.1. Synthesis and crystallization methods   

PR3C was synthesized following the procedure reported by So *et al.* (2018 ▸) and its chemical structure was confirmed by conventional analytical techniques. The crystallization of PR3C was carried out by liquid–liquid diffusion and the liquid–vapour diffusion method using a combination of various good (highly soluble) and poor (insoluble or less soluble) solvents (Table 1 ▸). The solvents used in this study were all commercially available and used without further purification.

### 2.2. Data collection and structure refinement details   

The diffraction data for PR3R were collected at 223 K on a Rigaku XtaLAB PRO diffractometer using graphite-monochromated Cu *K*α radiation (λ = 1.54187 Å). Data reduction was performed using *CrysAlis PRO* 1.171.39.20a (Rigaku Oxford Diffraction, 2015 ▸). The structure was solved by direct methods using *SHELXT* Version 2014/5 (Sheldrick, 2014 ▸) and refined by full-matrix least-squares methods based on *F* ^2^ using *SHELXL* Version 2014/7 (Sheldrick, 2015 ▸). All non-H atoms were refined anisotropically. The positions of all H atoms were calculated geometrically and estimated with the riding model. Structure analysis was performed using *CrystalStructure* 4.2 (Rigaku, 2017 ▸). The crystal structures were visualized and evaluated using *Mercury* 3.9 (Macrae *et al.*, 2008 ▸).

### 2.3. Differential scanning calorimetry (DSC) and powder X-ray diffraction (PXRD) measurements   

DSC measurements were conducted in crimped aluminium pans using a Rigaku Thermo Plus 2 DSC8230 at a heating rate of 10 K min^−1^. The typical weight of the sample was 1.9–2.0 mg. Before and after the DSC measurement, each powder sample was characterized by PXRD measurements, which were carried out using a Rigaku R-AXIS Rapid imaging-plate diffractometer with a graphite-monochromated Cu *K*α radiation (λ = 1.54187 Å) source at room temperature. Measurements were conducted in the 2θ range of 5–35°.

### 2.4. Calculation of the conformational energy   

The H-atom positions were initially optimized using *Mercury* 3.9 (Macrae *et al.*, 2008 ▸) in all calculations. The three polymorphs were optimized using the B3LYP (Lee *et al.*, 1988 ▸; Becke, 1993 ▸) functional coupled with the 6-31+G (d,p) basis set. The conformational energy was calculated using density functional theory (Frisch *et al.*, 2016 ▸) with *Gaussian16* at the ωB97X-D/6-31G(d) (Chai & Head-Gordon, 2008 ▸) level of theory. Single-point energy calculations were carried out using the atomic coordinates obtained by X-ray analysis.

### 2.5. Evaluation of intermolecular interactions   

The intermolecular interactions of the structures of the three polymorphs were evaluated by two different methods. Lattice energy calculations were performed using the atom–atom Coulomb–London–Pauli (AA-CLP) model, a computer program package for the empirical calculation of intermolecular interactions and crystal energies developed by Gavezzotti (2011 ▸). This module calculates the total lattice energy using the unit-cell parameters, space group and atomic coordinates. The H-atom positions were normalized using *Mercury* 3.9 (Macrae *et al.*, 2008 ▸) in all calculations. Hirshfeld surface analysis was also conducted using *CrystalExplorer* (McKinnon *et al.*, 2004 ▸). The calculation was carried out using the atomic coordinates from the crystal structure.

## 3. Results and discussion   

### 3.1. Crystallization of PR3C   

Crystallization was attempted considering three conditions: two crystallization methods, different solvent combinations and three different temperatures (278, 288 and 298 K). The concentration of the dye solution was fixed at 0.22 *M*. The results are summarized in Table 1 ▸. Crystallization runs 2, 4, 5 and 6 gave two polymorphs concomitantly, whereas runs 3 and 7 gave PR3Y; in contrast, runs 8, 10 and 11 gave only PR3O. Crystals of PR3R were not obtained alone in a single sample. Two different results were identified in crystallization run 1, which proceeded with the solvent combination of CHCl_3_ and *n*-hexane. In most samples, only PR3O was precipitated, and PR3R and PR3O were concomitantly obtained in relatively few samples. Different crystallization results obtained for the same solvent combination were also observed in the combination of CH_2_Cl_2_ and *n*-hexane (number 6). In this case, many samples showed concomitant formation of crystals of PR3O and PR3Y, and a small number of samples showed concomitant crystals of PR3R and PR3O. In the case of run number 9, the three polymorphs were obtained concomitantly. Notably, in all cases, the polymorphs were obtained concomitantly only at relatively low temperature (278 and 288 K). None of the obtained polymorphs changed to other forms at room temperature, even after being left for several months.

### 3.2. Geometrical comparisons of PR3R, PR3O and PR3Y   

**Figure 2:**
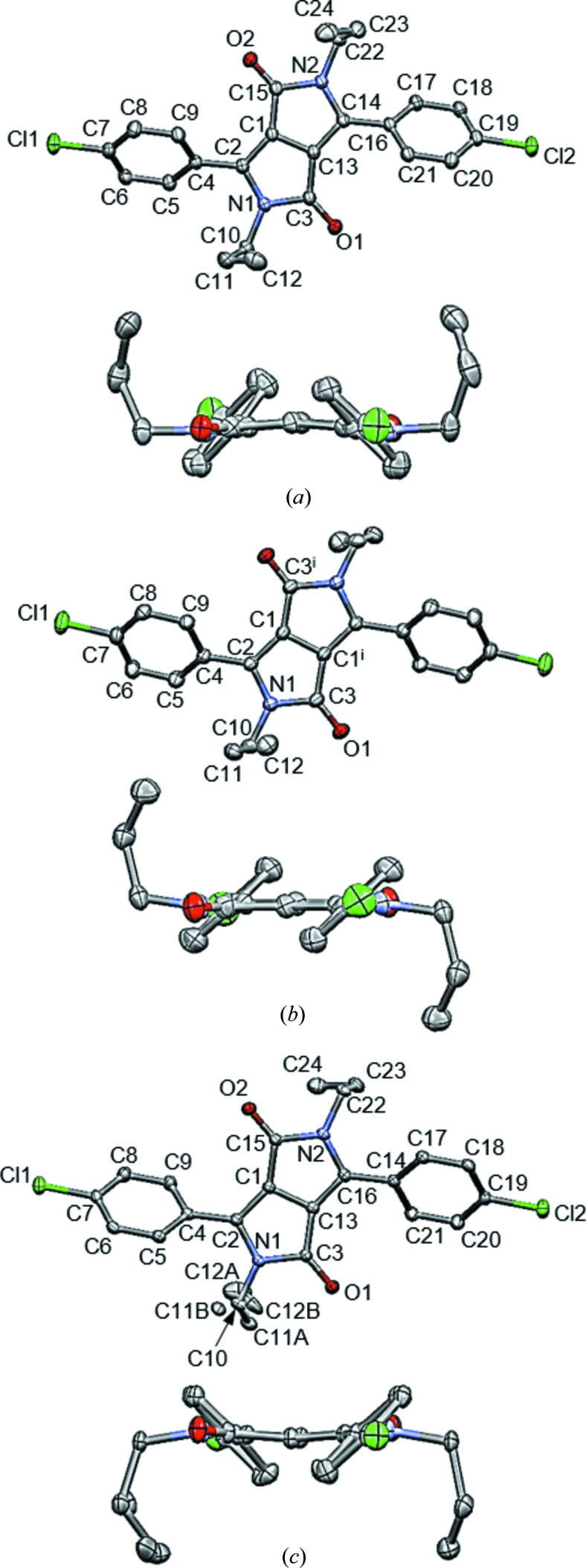
*ORTEP*-like diagrams of the molecular geometries showing displacement ellipsoids at the 30% probability level. H atoms are omitted for clarity. (*a*) PR3R, (*b*) PR3O, (*c*) PR3Y [viewpoint perpendicular to the DPP core (top) and viewpoint along the C1—C13 bond (bottom)].

**Figure 3:**
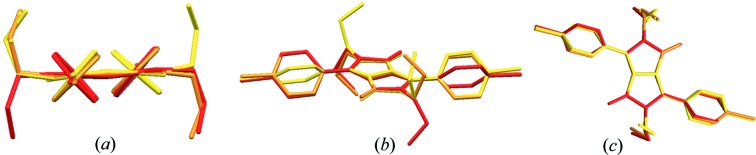
An overlay of the molecular geometries of the three polymorphs (red = PR3R, orange = PR3O, yellow = PR3Y (*A* set)]. Short (*a*) and long (*b*) molecular axes perspective views of the three polymorphs, and the viewpoint from the direction perpendicular to the DPP core (*c*).

Single-crystal X-ray analysis indicated that PR3R and PR3O crystals belong to the monoclinic *I*2/*a* and *P*2_1_/*c* space groups, respectively, while PR3Y crystallized in the triclinic space group *P* 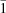 (Table 2 ▸). In PR3O, the molecule is located on an inversion centre whereby half of the molecule is in the asymmetric unit. As shown in Fig. 2 ▸, the three differently coloured polymorphs have distinct molecular conformations. The substituted propyl groups of PR3O are oriented towards opposite sides of the DPP core [Fig. 2 ▸(*b*)], whereas those of PR3R and PR3Y are oriented in the same direction. The overlay of the molecules of the three polymorphs reveals significant differences in conformation (Fig. 3 ▸).

#### 3.2.1. Molecular geometries   

**Figure 4:**
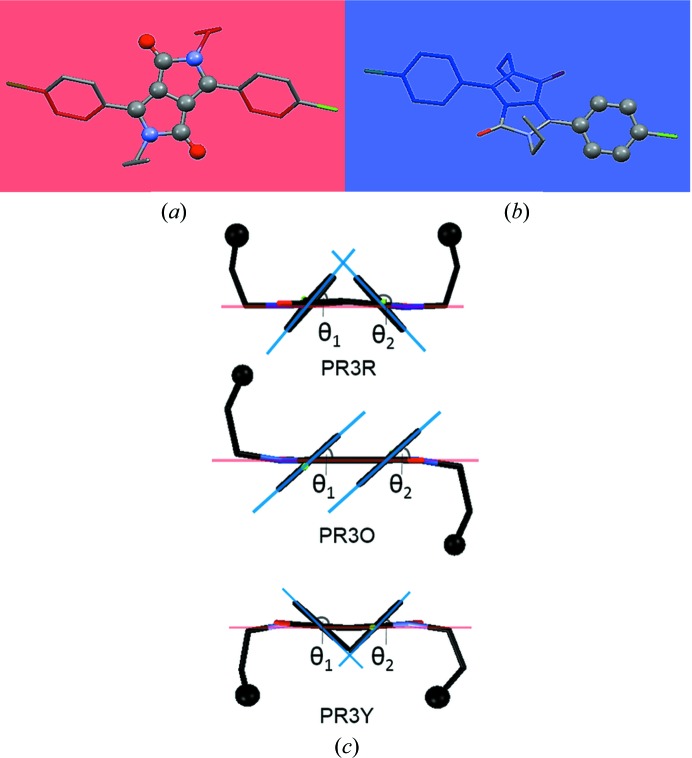
Geometric parameters used for the evaluation of the molecular geometry in the crystal structures of PR3R, PR3O and PR3Y: (*a*) plane I represents the DPP core consisting of ten atoms, (*b*) plane II represents the phenyl group consisting of six atoms, (*c*) the definition of the values listed in Table 3 ▸ used to evaluate the dihedral angle between the DPP core and the phenyl ring.

We have checked the molecular structure of the three polymorphs in detail, particularly focusing on both the planarity of the DPP core and the geometrical relationship between the DPP core, the phenyl rings and the propyl groups. The bond lengths in each polymorph show no particular differences in the dye chromophore with respect to bond alternation (Table S1 in the supporting information). In addition, among the three polymorphs, no significant differences were observed in the bond angles of the DPP core. For the evaluation of the planarity of the DPP core, the least-squares mean plane of a set of the ten component atoms of the DPP core of the three polymorphs was defined as plane I [Fig. 4 ▸(*a*)]. The distances between plane I and each constituent atom are listed in Table S2. PR3O has the most planar π-conjugated system, as shown by the small deviation of 0.003 (2) to 0.012 (2) Å. On the other hand, for PR3R and PR3Y, the deviation range was 0.000 (2)–0.105 (2) and 0.013 (2)–0.117 (2) Å, respectively, resulting in a slightly distorted shape of the core with the C1—C13 bond as the centre.

The characteristic molecular structure of each polymorph shown in Fig. 3 ▸ was also correlated with the geometrical relationship between the DPP core and the phenyl rings and propyl groups. The first was estimated from the dihedral angle between the DPP core and the phenyl rings, and the latter was examined to measure the torsion angles of the propyl groups. As shown in Fig. 4 ▸(*b*), the dihedral angle between the DPP core and the phenyl group was obtained by forming plane II, which contains six C atoms of the phenyl group in the least-squares mean plane. In addition, the torsion angles of the propyl groups were evaluated for N—C—C—C and C—N—C—C. The evaluation of the molecular geometry is summarized in Table 3 ▸. (The structure of PR3Y was divided into two sets due to the disorder of the propyl substituent.) We found that PR3R has the most twisted dihedral angle between planes I and II [50.65 (8) and 132.86 (8)°, respectively]. PR3O showed a less twisted value of 41.38 (11)°, and, in the case of PR3Y, the values were 43.95 (9)° and 132.71 (9)° [47.29(9) and 136.05(9) in Table 3], respectively. The torsion angles of the overall propyl group of the molecule in the three polymorphs (Table 3 ▸) were quite similar. For example, the torsion angles of PR3R, PR3O and both *A* and *B* sets of PR3Y have quite similar values even if some show the opposite sign. One exception could be found in the *A* set of PR3Y, showing a significantly different magnitude in C2—N1—C10—C11 and C3—N1—C10—C11, and in some cases the opposite sign was also found. The characteristics of the rotatable phenyl ring and flexible alkyl substituent of these three polymorphs imply that a different arrangement of molecules by the introduction of a flexible alkyl substituent plays an important role in the occurrence of conformational polymorphism in DPP, unlike the case where the unsubstituted DPP exhibits a rigid planar structure (Mizuguchi, 2000 ▸). Unsubstituted DPP is known to have two polymorphs with different pigment properties (Ciba-Geigy Corporation, 1983 ▸, 1997 ▸, 1999 ▸). Crystal structure analysis has only been reported for its α-form (Mizuguchi *et al.*, 1992 ▸) and the β-form was only identified by its powder X-ray pattern. In the reported crystal structure of unsubstituted DPP, the DPP molecules have a small dihedral angle [7 (1)°] between the DPP core and the phenyl ring, resulting in an almost planar molecular structure. This molecular structure was correlated with its characteristic brick-wall crystal structure formed via intermolecular hydrogen bonds and π–π interactions (Mizuguchi *et al.*, 1992 ▸). This structural property characterized by the planar molecular conformation and intermolecular interactions including hydrogen bonds and π–π interactions was also recognized in the reported crystal structure of *para*-chlorinated DPP (Mizuguchi *et al.*, 1993 ▸).

#### 3.2.2. Crystal structures   

**Figure 5:**
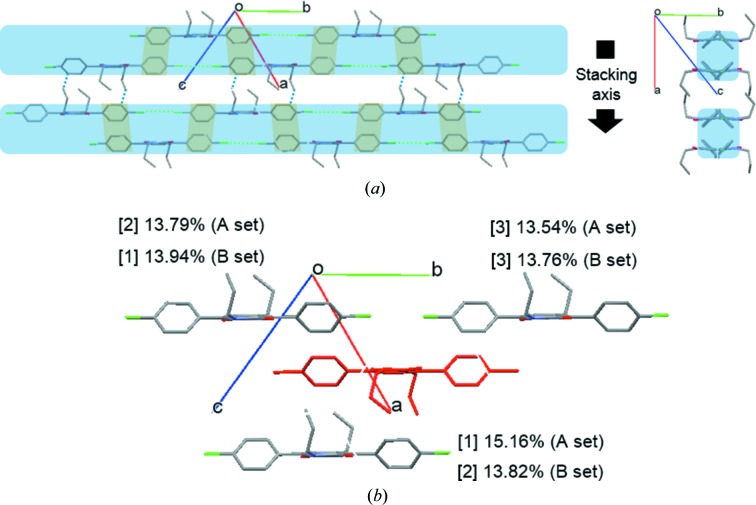
The molecular packing structure of PR3Y (*a*) showing the one-dimensional columns (blue box) formed by π–π interactions (orange box), the interactions between chains formed by halogen-bonding interactions (green dotted line) and the CH⋯π interactions (blue dotted line) between the columns (left). Packing of molecules viewed along the [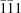] direction (right). (*b*) A pair of molecules on the (011) plane that contribute the most significantly to the lattice stabilization. The lattice energy was calculated by dividing into *A* and *B* sets due to the disorder of one of the propyl substituents. The red molecule represents the reference molecule.

**Figure 6:**
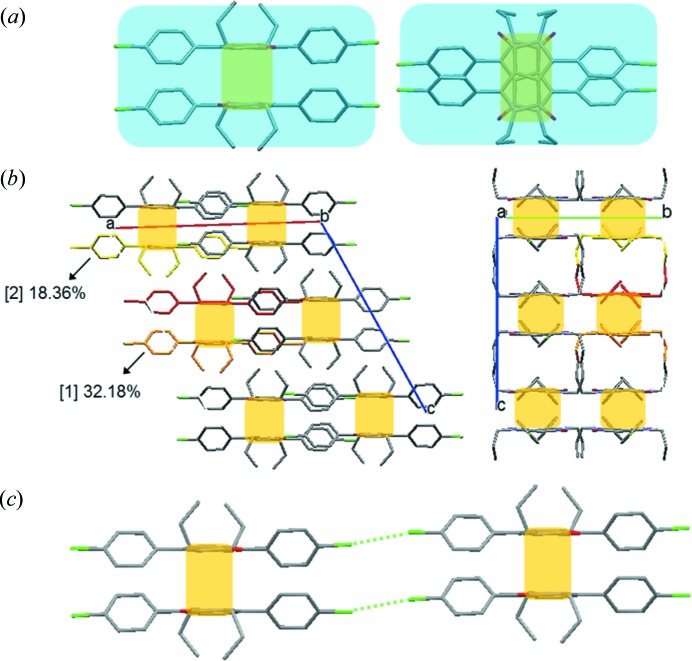
(*a*) Molecular pair (blue box) of PR3R with π–π interactions (orange box) between the DPP core [viewpoint along the DPP core (left) and viewpoint perpendicular to the DPP core (right)]. (*b*) A one-dimensional column consisting of molecular pairs along the *c* axis [viewpoint along the *b* axis (left) and that along the *a* axis (right)]. The red molecule represents the reference molecule. (*c*) The green dotted line indicates the intermolecular halogen-bonding interaction between two molecular pairs.

**Figure 7:**
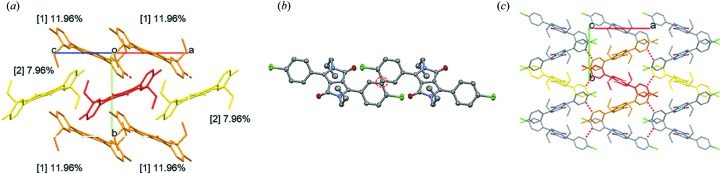
(*a*) Molecular arrangement of PR3O viewed parallel to the (101) plane. (*b*) Overlap of two molecules for PR3O (red dotted circle). (*c*) The herringbone-like stacking arrangement of PR3O along the *b* axis (red dotted line represents interaction between two C atoms). The red molecule in (*a*) and (*c*) represents the reference molecule.

The molecular packing behaviour of the three polymorphs was analysed using lattice energy calculations. In the crystal structure of PR3Y, a chain structure is present, formed by weak intermolecular halogen-bonding interactions having a distance of 3.4797 (9) Å along the long molecular axis. The chain structures interact via π–π interactions between the two phenyl moieties of the adjacent molecules at a distance of 3.339 (2) Å, thus forming a one-dimensional column [Fig. 5 ▸(*a*)]. In addition, there are two columnar structures interacting via C—H⋯π interactions [blue dotted line in Fig. 5 ▸(*a*)]. The lattice energy calculation of PR3Y was achieved using the AA-CLP model and dividing the structure into two sets (*A* set and *B* set) because of the disorder of one of the propyl substituents. As shown in Fig. 5 ▸(*b*) and Table 4 ▸, adjacent molecules along the stacking axis in both sets contributed to the stabilization of the lattice. The third energy contribution of PR3Y is similar to the first and second energy contributions; this is a unique result found only in PR3Y. All of these contributions to the lattice stabilization energy were found to be related to the π–π interactions between the reference and the adjacent molecule and not related to the halogen-bonding interactions along the long molecular axis [Fig. 5 ▸(*b*)]. The crystal structure of PR3R is shown in Fig. 6 ▸. The molecules of PR3R form a molecular pair between two adjacent molecules with π–π interactions [3.343 (1) Å] of the DPP core [Fig. 6 ▸(*a*)]. The molecules in these pairs also showed several short contacts (O2⋯H17, O1⋯H5 and O1⋯H10*B*). In the crystal structure of PR3R, the molecular pairs formed a one-dimensional column through short contacts with each other along the *c* axis [Fig. 6 ▸(*b*)], and these columns interact with each other by Cl⋯Cl intermolecular halogen-bonding interactions [3.544 (1) Å] along the long molecular axis [Fig. 6 ▸(*c*)]. This packing behaviour is consistent with the results of the lattice energy calculations, indicating that the orange molecule that forms a molecular pair with the red reference molecule makes the largest contribution (32.2%) to the stabilization of the lattice, as shown in Fig. 6 ▸(*b*) and Table 4 ▸. The yellow molecule of the adjacent molecular pair from the red reference molecule was also found to contribute to stabilization by a second contribution (18.37%) along the *c* axis. In the crystal structures of PR3Y and PR3R, as described above, a directional halogen-bonding interaction was observed along the long molecular axis, whereas PR3O showed a completely different crystal structure because of the different molecular arrangement. In the crystal structure of PR3O, the red reference molecule in Fig. 7 ▸(*a*) interacts with the four adjacent orange molecules through short contacts such as C12⋯C3, O1⋯H5 and H12*C*⋯H10*B*. In addition, a van der Waals contact between two C atoms (C8⋯C8) of the phenyl rings was found in two adjacent yellow molecules with respect to the reference molecule [Fig. 7 ▸(*b*)]. As shown in Fig. 7 ▸(*c*), PR3O showed a herringbone-like stacking arrangement along the *b* axis, unlike the other polymorphs. The lattice energy calculation of PR3O also showed that the first (11.95%) and second (7.96%) energetic contributions help to stabilize the crystal lattice [Fig. 7 ▸(*a*) and Table 4 ▸].

The present results indicate that these three polymorphs have different crystal structures, and the π–π stacked molecule pairs make a major contribution to their lattice stabilization. However, directional halogen-bonding interactions were also found to contribute to the structural diversity.

**Figure 8:**
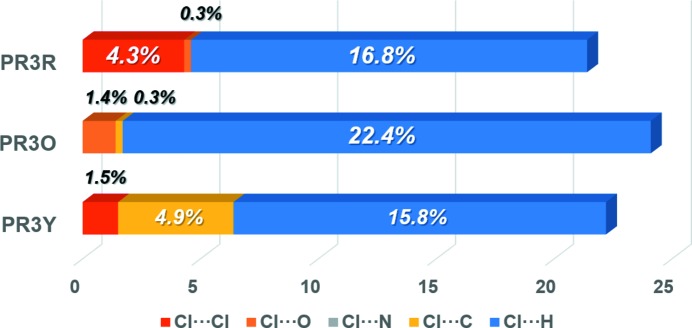
Relative contributions of the halogen-bonding interactions to the Hirshfeld surface area of the three polymorphs.

The intermolecular halogen-bonding interactions (Cl⋯Cl, Cl⋯O, Cl⋯N, Cl⋯C and Cl⋯H) in the crystal structures of PR3R, PR3O and PR3Y were quantified by Hirshfeld surface analysis. A bar chart of the percentage contributions of various intermolecular halogen-bonding interactions for all the investigated polymorphs is shown in Fig. 8 ▸. For the Cl contacts, the contribution of direct halogen Cl⋯Cl contacts was determined for the crystal structures of PR3R (4.3%) and PR3Y (1.5%) with the exclusion of the terminal H atoms. These values are consistent with the crystal structures of PR3R and PR3Y, which show directional intermolecular interactions along the long molecular axis. On the other hand, the contribution of the Cl⋯C interactions was found to be about 5% in PR3Y, and this value is larger than in the other polymorphs. This feature is attributed to the presence of C—Cl⋯π halogen contacts [3.582 (2) and 3.597 (2) Å for Cl1⋯C1 and Cl1⋯C2, respectively], which are longer than the sum of the van der Waals radii (3.45 Å). In the case of PR3O, which has a herringbone structure, the above-mentioned contribution to the intermolecular halogen-bonding interactions was hardly observed. These results suggest that different intermolecular interactions, especially the contribution of the diverse intermolecular halogen-bonding interactions in these polymorphs, contribute to the formation of the characteristic crystal structure of each polymorph.

### 3.3. Thermal stability of three polymorphs   

#### 3.3.1. DSC investigations   

For all the polymorphic forms, a very weak broad peak around 380 K was observed in the DSC measurement. This event might be related to the removal of a negligible amount of residual solvent. On heating, PR3R and PR3O showed another endothermic peak that corresponds to their melting point at 505.8 and 508.3 K, respectively. The DSC thermogram of PR3Y contains two more endothermic peaks at 450 and 506.9 K. The second signal was intense and corresponds to the melting point, whereas the first peak is relatively broad, corresponding to a polymorphic phase transition. This polymorphic phase transition indicates the thermo­salient effect accompanied by mechanical behaviour, and the PXRD result of PR3Y right after the phase transition coincided with the results obtained for PR3O (So *et al.*, 2018 ▸). This suggests that PR3O is thermodynamically more stable after the phase transition and the two polymorphs are in an enantiotropic relationship with each other. This is also consistent with the fact that the melting point of PR3O, which is thermodynamically more stable at high temperatures, is higher than that of PR3Y.

#### 3.3.2. Thermodynamic stability relationships between the three polymorphs   

**Figure 9:**
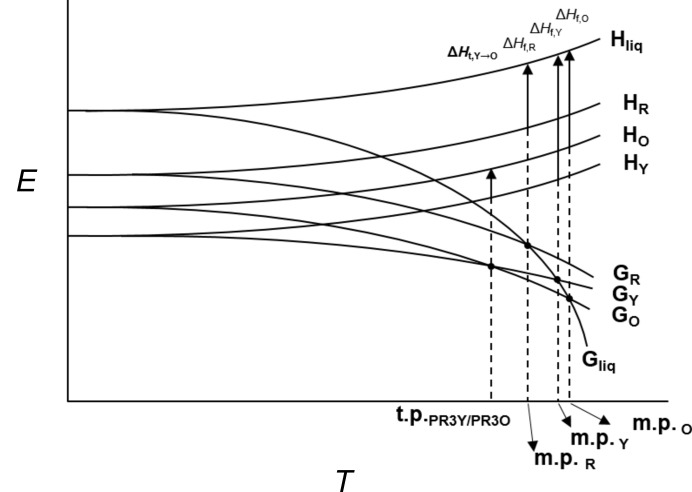
Semi-empirical diagram of energy versus temperature of PR3R, PR3O and PR3Y. *G* is the Gibbs free energy and *H* is the enthalpy. R, O and Y represent PR3R, PR3O and PR3Y, respectively. In addition, t.p. is the transition point between the two polymorphs and m.p. is the melting point.

The results of the DSC measurement clearly demonstrate the thermodynamic stability relationship between PR3O and PR3Y (Fig. S1). These two polymorphs are enantiotropically related to each other based on the heat of transition rule (Burger & Ramberger, 1979 ▸), which means that PR3Y is more stable at lower temperatures, whereas PR3O is more stable after the transition point. The DSC profile of PR3R showed the lowest enthalpy of fusion compared with the other two polymorphs. Based on the heat of fusion rule or the entropy of fusion rule, PR3R is thermodynamically metastable and monotropically related to the other two polymorphs. The enthalpy of fusion of PR3Y showed the largest value and PR3O showed the next largest value. Moreover, the calculated total energy, defined as the sum of the relative conformational energy and the lattice energy, was also lower in both disordered forms of PR3Y than in PR3R and PR3O (Table 5 ▸). This is also supported by the fact that the corresponding stable crystal form of PR3Y has a higher density than the metastable forms PR3R and PR3O because of the efficient packing structure (Bernstein, 2002 ▸). It has long been thought that the polymorphic form with the highest density is the thermodynamically stable form. However, as an exception to this correlation, it has been discussed that an energetically favourable hydrogen-bond-dominated packing arrangement can lead to low-density crystal structures in a polymorphic system (Nelyubina *et al.*, 2010 ▸; Ng *et al.*, 2014 ▸). Because the three polymorphs discussed in this study did not show any strong hydrogen bonds in the crystal structure, it is reasonable to infer that PR3Y is thermodynamically more stable than the other two. From the above description, we could draw a semi-schematic energy diagram of the thermodynamic relationship between PR3R, PR3O and PR3Y (Fig. 9 ▸).

To comprehend the thermodynamic relationship between the three polymorphs, we used the method of Yu (1995 ▸), where the thermal stability of two polymorphs can be identified from the melting data, which makes it possible to confirm the relationship between the two polymorphs, that is, monotropic or enantiotropic. When Δ*H*_0_ > 0, the relationship between two phases is enantiotropic, and a monotropic relationship is expected when Δ*S*_0_ < 0. As a result, the values obtained by calculation are in agreement with a previous expectation (Table S2). In addition, the transition temperature between PR3Y and PR3O, which are enantiotropically related, was calculated from Δ*H*_0_/Δ*S*_0_ to be 459.8 K, which is in good agreement with the transition point observed in the DSC profile (450 K).

## 4. Conclusion   

The crystal structures of three polymorphic forms of chlorinated DPP with propyl substituents were characterized, and lattice energy calculation and Hirshfeld surface analysis were carried out. All polymorphic forms showed different molecular conformations, as well as crystal structures. In the analysed crystal structures, PR3R and PR3Y showed intermolecular interactions with directional Cl⋯Cl interactions, whereas PR3O showed a herringbone packing structure without any specific intermolecular interactions. This result suggests that the packing effect by different arrangements of the surrounding molecules seems to play an important role in controlling polymorphism in the DPP chromophore. Although we have not discussed the differences in colour between the three polymorphs in this paper, as a result of preliminary molecular orbital calculations based on time-dependent density functional theory using the coordinates of the molecule, there was no significant difference in the absorption characteristics in the three polymorphs. Therefore, the difference in their colours needs to be characterized proceeding with examination of the intermolecular interactions including fluorescence properties, and this is now in progress.

The thermodynamic relationship was also determined by thermal analysis and theoretical methods; PR3Y is thermodynamically the most stable form at temperatures before the transition point, whereas PR3O is the most stable form at temperatures after the transition point. PR3R was found to be a monotropic metastable form.

PR3Y has already been reported to exhibit a thermosalient effect in the transition to PR3O. Existing compounds showing thermosalient effects exhibit phase transitions to high-temperature polymorphic phases with clear anisotropic changes to the crystal but without changes in the space group. However, PR3Y differs from the conventional thermosalient crystals in that PR3O, which is a high-temperature phase of PR3Y, was obtained at the time of crystallization and converted to a completely different crystal structure, as well as space group, during the phase transition. Because the dynamic behaviour arising from the sudden release of energy made it difficult to obtain single-crystal X-ray diffraction data after the phase transition of PR3Y, further structural analysis based on temperature-dependent PXRD measurements is required to obtain a clear understanding of this phenomenon.

## Supplementary Material

Crystal structure: contains datablock(s) global, I. DOI: 10.1107/S2052520619004773/bm5110sup1.cif

Structure factors: contains datablock(s) I. DOI: 10.1107/S2052520619004773/bm5110Isup3.hkl

Supporting information file. DOI: 10.1107/S2052520619004773/bm5110sup2.pdf

CCDC reference: 1880254

## Figures and Tables

**Table 1:** Various crystallization conditions and the polymorphs obtained In parentheses is given the types of concomitant polymorphs obtained. Run No. 6 resulted in two different combinations of concomitant polymorphs.

					No. of samples (obtained, tried)		
Method	No.	Good solvent	Poor solvent	Outcome of crystallization	278 K	288 K	298 K
Liquid–liquid diffusion	1†	CHCl_3_	*n*-Hexane	Only PR3O obtained	4, 9	2, 3	3, 3
				(PR3R and PR3O)	5, 9	1, 3	0, 3
	2	CHCl_3_	Ethanol	(PR3R and PR3Y)	15, 15	0, 3	0, 3
	3	CHCl_3_	*c*-Hexane	Only PR3Y obtained	3, 5	0, 5	0, 5
	4	Toluene	*n*-Hexane	(PR3O and PR3Y)	15, 15	0, 3	0, 3
	5	Benzene	*n*-Hexane	(PR3R and PR3O)	15, 15	0, 3	0, 3
	6	CH_2_Cl_2_	*n*-Hexane	(PR3O and PR3Y)	6, 9	3, 6	0, 3
				(PR3R and PR3O)	3, 9	3, 6	0, 3
	7	CH_2_Cl_2_	Ethanol	Only PR3Y obtained	3, 5	0, 5	0, 5
	8	CH_2_Cl_2_	*c*-Hexane	Only PR3O obtained	2, 5	1, 5	0, 5
Liquid–gas diffusion method	9	CHCl_3_	*n*-Hexane	(PR3R, PR3O and PR3Y)	6, 9	0, 3	0, 3
	10	Toluene	*n*-Hexane	Only PR3O obtained	5, 5	5, 5	5, 5
	11	Benzene	*n*-Hexane	Only PR3O obtained	5, 5	5, 5	5, 5

† Represents two cases obtained with concomitant polymorphs and only one single polymorph.

**Table 2:** Crystallographic details The crystal structures of PR3O and PR3Y have been published previously (So *et al.*, 2018 ▸).

	PR3R	PR3O	PR3Y
Crystal data			
*M*_r_	441.36	441.36	441.36
Crystal system, space group	Monoclinic, *I*2/*a*	Monoclinic, *P*2_1_/*c*	Triclinic, *P* 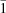
Temperature (K)	223	293	223
*a*, *b*, *c* (Å)	18.1075 (2), 14.16642 (16), 18.5436 (3)	11.31549 (16), 9.66903 (15), 9.86401 (15)	9.7334 (2), 9.9244 (2), 12.4910 (3)
α, β, γ (°)	90, 117.163 (2), 90	90, 96.4687 (13), 90	89.585 (2), 69.561 (2), 67.774 (2)
*V* (Å^3^)	4232.16 (12)	1072.35 (3)	1035.74 (4)
*Z*, *Z*′	8, 1	2, 0.5	2, 1
*D*_calc_ (g cm^−3^)	1.385	1.367	1.415
μ (mm^−1^)	2.95	2.91	3.01
Crystal size (mm)	0.35 × 0.12 × 0.10	0.65 × 0.36 × 0.20	0.29 × 0.14 × 0.06

Data collection			
Diffractometer	Rigaku XtaLAB P200	Rigaku XtaLAB P200	Rigaku XtaLAB P200
*T*_min_, *T*_max_	0.410, 0.753	0.174, 0.564	0.644, 1.000
No. of measured, independent and observed reflections	10850, 3726, 3303 [*F* ^2^ > 2.0σ(*F* ^2^)]	5661, 1868, 1672 [*F* ^2^ > 2.0σ(*F* ^2^)]	9651, 3580, 3182 [*I* > 2σ(*I*)]
*R*_int_	0.034	0.032	0.027
(sin θ/λ)_max_ (Å^−1^)	0.595	0.595	0.595

Refinement			
*R*[*F* ^2^ > 2σ(*F* ^2^)], *wR*(*F* ^2^), *S*	0.046, 0.139, 1.10	0.050, 0.142, 1.07	0.042, 0.122, 1.06
No. of reflections	3726	1868	3580
No. of parameters	271	136	292
Δρ_max_, Δρ_min_ (e Å^−3^)	0.35, −0.25	0.27, −0.42	0.44, −0.25

**Table 3:** Results of the molecular geometry (°) evaluations of the crystal structures of PR3R, PR3O and PR3Y The structure of PR3Y was divided into two sets (*A* set and *B* set) due to the disorder of the propyl substituent.

	PR3R	PR3O	PR3Y
θ_1_	50.65 (8)	41.38 (11)	136.05 (9)
θ_2_	132.86 (8)	41.38 (11)	47.29 (9)
N1—C10—C11—C12	−61.9 (3)	55.4 (2)	−53.2 (5) (*A* set)
			60 (1) (*B* set)
N2—C22—C23—C24	−60.0 (3)	55.4 (2)	63.9 (3)
C2—N1—C10—C11	−66.8 (3)	66.4 (2)	100.7 (2) (*A* set)
			58.6 (*B* set)
C3—N1—C10—C11	106.6 (2)	−99.3 (2)	−61.4 (2) (*A* set)
			−103.5 (4) (*B* set)
C14—N2—C22—C23	−65.4 (3)	66.4 (2)	66.9 (2)
C15—N2—C22—C23	102.8 (2)	−99.3 (2)	−94 (2)

**Table 4:** Total energy of all molecular pairs and the contributions of the major molecular pairs

	Total energy† (kJ mol^−1^)	Most stable pair (kJ mol^−1^)	Second stable pair (kJ mol^−1^)	Third stable pair (kJ mol^−1^)
PR3R	−182.6	−117.6 (32.2%)	−67.1 (18.37%)	−20.2 (5.53%)
PR3O	−176.5	−42.2 (11.95%)	−28.1 (7.96%)	−13.6 (3.85%)
PR3Y *A* set	−200.4	−60.8 (15.16%)	−55.3 (13.79%)	−54.3 (13.54%)
PR3Y *B* set	−197.9	−55.2 (13.94%)	−54.6 (13.79%)	−54.5 (13.76%)

† The total energy of all molecular pairs is the summation of the energies of all calculated molecular pairs. Half of the total energy corresponds to the lattice energy.

**Table 5:** Total crystal energies of PR3R, PR3O and PR3Y

	Lattice energy† (kJ mol^−1^)	Relative conformational energy‡ (kJ mol^−1^)	Total energy§ (kJ mol^−1^)
PR3R	−182.6	0.0005	−182.59
PR3O	−176.5	3.24	−173.25
PR3Y *A* set	−200.4	1.67	−198.72
PR3Y *B* set	−197.9	0	−197.90

† Lattice energy was calculated using the AA-CLP model.

‡ Relative conformational energy is the energy difference from the conformational energy of the lowest-energy forms.

§ Total energy was estimated from the sum of the lattice energy and the relative energy.
