# Supplementary material for: Three differently coloured polymorphs of 3,6-bis­(4-chloro­phenyl)-2,5-di­propyl-2,5-di­hydro­pyrrolo­[3,4-c]pyrrole-1,4-dione
Source: Acta Crystallogr B Struct Sci Cryst Eng Mater. 2019 May 23;75(Pt 3):414–22. doi: 10.1107/S2052520619004773 (PMC6549220; doi:10.1107/S2052520619004773)
Supplement: Supplementary file 3 [file b-75-00414-sup2.pdf]

Acta Cryst B

STRUCTURAL SCIENCE
CRYSTAL ENGINEERING
MATERIALS

Volume 75 (2019)

Supporting information for article:

Three differently coloured polymorphs of 3,6-bis(4-chlorophenyl)-2,5-dipropyl-2,5-dihydropyrrolo[3,4-c]pyrrole-1,4-dione

Hee-Soo So and Shinya Matsumoto

Three differently coloured polymorphs in 3,6-bis(4-chlorophenyl)-2,5-dipropyl-2,5-dihydropyrrolo[3,4-c]pyrrole-1,4-dione

Hee-Soo So and Shinya Matsumoto*

Table S1 Bond lengths and bond angles for the DPP core of three polymorphs.

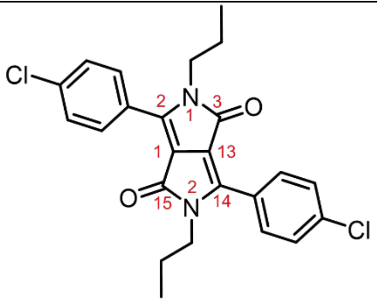

Bond lengths [Å]						Bond angles [°]					
No.	Atom 1	Atom 2	**PR3R**	**PR3O**	**PR3Y**	Atom 1	Atom 2	Atom 3	**PR3R**	**PR3O**	**PR3Y**
1	N1	C3	1.429(3)	1.424(2)	1.423(2)	C1	C2	N1	107.6(2)	107.7(1)	107.7(2)
2	N1	C2	1.393(2)	1.390(2)	1.389(3)	C2	N2	C3	111.3(1)	111.1(1)	111.5(2)
3	N2	C15	1.431(3)	1.424(2)	1.428(2)	N1	C3	C13	103.7(1)	104.0(1)	104.0(7)
4	N2	C14	1.391(2)	1.390(2)	1.387(2)	C3	C13	C1	108.5(2)	108.1(1)	107.5(2)
5	C15	C1	1.447(2)	1.446(2)	1.456(2)	C13	C1	C2	108.9(2)	109.1(1)	109.3(2)
6	C13	C14	1.378(3)	1.373(2)	1.375(2)	C1	C13	C14	109.0(2)	109.1(1)	108.9(2)
7	C13	C1	1.423(3)	1.421(2)	1.430(3)	C13	C14	N2	107.6(2)	107.7(1)	108.0(2)
8	C13	C3	1.446(2)	1.446(2)	1.457(2)	C14	N2	C15	111.3(1)	111.1(1)	111.4(2)
9	C1	C2	1.377(3)	1.373(2)	1.375(2)	N2	C15	C1	103.6(1)	104.0(1)	103.7(2)
10						C15	C1	C13	108.4(2)	108.1(1)	107.9(2)

Table S2 Deviations (Å) of the ten component atoms of the DPP core from their least-squares mean planes in the crystal structures of **PR3R**, **PR3O**, and **PR3Y**.

**PR3R**		**PR3O**		**PR3Y**	
C1	0.103 (2)	C1	0.003 (2)	C1	0.117 (2)
C2	0.001 (2)	C2	0.012 (2)	C2	0.036 (2)
C3	0.002 (2)	C3	0.008 (2)	C3	0.014 (2)
N1	0.078 (1)	N1	0.010 (1)	N1	0.045 (2)
C13	0.105 (2)	O1	0.009 (2)	C13	0.117 (2)
C14	0.003 (2)			C14	0.033 (2)
C15	0.000 (2)			C15	0.013 (2)
N2	0.076 (1)			N2	0.043 (2)
O1	0.026 (1)			O1	0.093 (2)
O2	0.026 (1)			O2	0.095 (2)

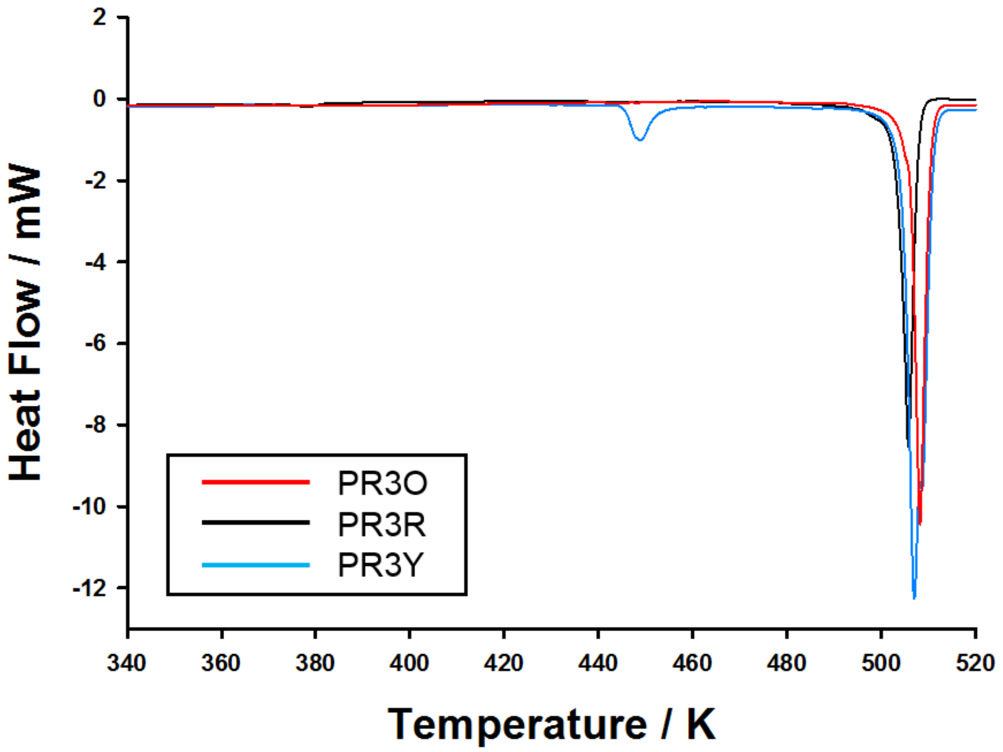

Figure S1 DSC profiles of PR3R, PR3O, and PR3Y. PR3Y shows an endothermic peak at 450 K, which corresponds to the thermosalient effect. In the temperature range of 506–508 K, the peaks correspond to their melting points.

Table S3 Calculated values for the thermodynamic properties of all polymorphs based on Yu's method [1].

	ΔH_0 /kJ mol^{-1}	ΔS_0/J mol^{-1} K^{-1}	Transition point (K)
**PR3O → PR3R**	-0.9568	-0.0017	559.1 (monotropic)
**PR3Y → PR3O**	0.5002	0.0010	459.8 (enantiotropic)
**PR3Y → PR3R**	-1.4557	-0.0028	520.5 (monotropic)

[1] Yu, L. (1995). *J. Pharm. Sci.* **84**, 966–974.
